# Grape-Leaf Extract Attenuates Alcohol-Induced Liver Injury via Interference with NF-κB Signaling Pathway

**DOI:** 10.3390/biom10040558

**Published:** 2020-04-06

**Authors:** Yhiya Amen, Asmaa E. Sherif, Noha M. Shawky, Rehab S. Abdelrahman, Michael Wink, Mansour Sobeh

**Affiliations:** 1 Department of Pharmacognosy, Faculty of Pharmacy, Mansoura University, Mansoura 35516, Egypt; asmaasherif80@yahoo.com; 2 Department of Pharmacognosy, College of Pharmacy, Prince Sattam Bin Abdulaziz University, Al-Kharj 11942, Saudi Arabia; 3 Department of Pharmacology and Toxicology, Faculty of Pharmacy, Mansoura University, Mansoura 35516, Egypt; noha.shawki.elsayed@gmail.com; 4 Department of Pharmacology and Toxicology, College of Pharmacy, Taibah University, Al-Madina Al-Munawwarah 30001, Saudi Arabia; 5 Institute of Pharmacy and Molecular Biotechnology, Heidelberg University, Im Neuenheimer Feld 364, 69120 Heidelberg, Germany; mansour.ahmed98@yahoo.com; 6 AgroBioSciences Research Division, Mohammed VI Polytechnic University, Lot 660–Hay Moulay Rachid, Ben-Guerir 43150, Morocco

**Keywords:** grape-leaf extract, oxidative stress, NF-κB, apoptosis, LC-MS, liver injury, antioxidants

## Abstract

Grape (*Vitis vinifera*) leaf extracts (GLEs) are known to be rich in phenolic compounds that exert potent antioxidant effects. Given the vulnerability of the liver to oxidative damage, antioxidants have been proposed as therapeutic agents and coadjuvant drugs to ameliorate liver pathologies. The current study was designed to characterize secondary metabolites and investigate the hepatoprotective effects of GLE and its underlying mechanisms. The secondary metabolites were profiled using HPLC–PDA–ESI-MS, and forty-five compounds were tentatively identified. In experimental in vivo design, liver injury was induced by oral administration of high doses of ethanol (EtOH) for 12 days to male Sprague Dawley rats that were split into five different groups. Blood samples and livers were then collected, and used for various biochemical, immunohistochemical, and histopathological analyses. Results showed that GLE-attenuated liver injury and promoted marked hepatic antioxidant effects, in addition to suppressing the increased heat-shock protein-70 expression. Moreover, GLE suppressed EtOH-induced expression of nuclear factor-κB (NF-κB) p65 subunit and proinflammatory cytokine tumor necrosis factor-α. Caspase-3 and survivin were enhanced by EtOH intake and suppressed by GLE intake. Finally, EtOH-induced histopathological changes in liver sections were markedly normalized by GLE. In conclusion, our results suggested that GLE interferes with NF-κB signaling and induces antioxidant effects, which both play a role in attenuating apoptosis and associated liver injury in a model of EtOH-induced liver damage in rats.

## 1. Introduction

Different parts of the common grape (*Vitis vinifera* L., Vitaceae) are commonly used to produce wine and dietary antioxidant supplements with vitamins and minerals. The fresh leaves have also been traditionally used for various ailments, in addition to being considered as an important food source [1]. Grape-leaf extracts (GLEs) are known to be rich in phenolic compounds that were found to exert potent antioxidant effects [2,3,4,5]. Given the vulnerability of the liver to oxidative damage, the use of antioxidants has been proposed as therapeutic agents, as well as drug coadjuvants, to ameliorate liver pathologies. For instance, the fruit and seed extract showed hepatoprotective effects in liver injuries associated with immunology, infections, and drug intoxication [6]. Importantly, the hepatoprotective effects of GLE were demonstrated in a model of CCl_4_-induced acute liver damage, but the underlying mechanisms were not explored [1].

Alcohol, as a hepatotoxin, causes hepatic injury via alcohol-metabolism-induced oxidative stress, the formation of mutagenic acetaldehyde, and proinflammatory cytokine production. Moreover, increased alcohol intake accelerates its metabolism via cytochrome P450 (2E1) that, in turn, promotes further oxidative stress and reactive-oxygen-species (ROS) generation with further lipid peroxidation and alteration of hepatic antioxidant defenses, particularly reduced glutathione (GSH) [7]. Alcohol consumption is also associated with imbalanced immune responses that result in the increased production of proinflammatory cytokines, particularly tumor necrosis factor-α (TNF-α) after nuclear factor-κB (NF-κB) activation [8]. TNF-α and oxidative stress contribute to hepatic steatosis (fat deposition) and hepatitis (inflammation) that constitute alcoholic liver disease (ALD), one of the earliest signs of liver injury. Fatty liver is one of the most prevalent types of ALDs, in which more than 90% of all heavy drinkers develop fatty liver, while about 25% develop the more severe alcoholic hepatitis [9].

In the current study, we hypothesized that GLE could attenuate ethanol (EtOH)-induced liver injury. Therefore, we aimed to investigate the hepatoprotective effects of GLE and the underlying molecular mechanisms using a rat model in which multiple high oral doses of EtOH were administered for the short term to mimic binge-drinking behavior in humans. We also aimed at characterizing the secondary metabolites of in vivo tested GLE.

## 2. Material and Methods

### 2.1. Reagents and Chemicals

Sulphanilamide, vanadium chloride (VCl_3_), zinc sulphate (ZnSO_4_), pyrogallol, tris base, Ellman’s reagent, trichloroacetic acid (TCA), sodium dodecyl sulphate, thiobarbituric acid (TBA), 1, 1′, 3, 3′-tetramethoxypropane were purchased from Sigma Aldrich Chemical Co. (St. Louis, MO, USA). Reduced glutathione was purchased from Alpha Chemika (Mumbai, India), and methanol and absolute ethanol from El-Nasr for Pharmaceutical Chemicals (Egypt). All other chemicals used in the study were of high analytical grade.

### 2.2. Plant Material

The dried leaves of *Vitis vinifera* L. were obtained from Mansoura University Gardens (31°02′38.4″ N 31°21′03.5″ E.), Mansoura, Egypt in October 2017. The plant identity was confirmed by staff members at Department of Pomology, Faculty of Agriculture, Mansoura University. A voucher specimen (V-10-17) was deposited at the Department of Pharmacognosy, Faculty of Pharmacy, Mansoura University.

### 2.3. Extract Preparation

The freshly cut leaves were thoroughly washed with distilled water, shade-dried, and ground. The dried and powdered leaves (500 g) were extracted with 90% methanol at room temperature (2 L × 6 times). The combined methanol extract was evaporated to dryness under reduced pressure to give a total methanol extract (GLE) 76.32 g (yield 15.26%).

### 2.4. LC Analysis

Thermo Finnigan (Thermo Electron Corporation, Austin, TX, USA) LC system coupled with the mass spectrometer (LCQ-Duo ion trap) having an ESI source (ThermoQuest) was used. A Discovery HS F5 column (15 cm × 4.6 mm ID, 5 µm particles, Sigma-Aldrich Co, Steinheim, Germany) was used. The mobile phases were water and acetonitrile (ACN) (0.1% formic acid each). At 0 min, ACN was 5%, then increased to 30% over 60 min. The instrument was controlled by Xcalibur software (Xcalibur^TM^ 2.0.7, Thermo Scientific, Waltham, MA, USA). The MS was operated in negative mode, and ions were detected within 50–2000 *m*/*z* mass range in a full scan mode as before [10].

### 2.5. Experiment Animals

Male Sprague Dawley rats (~7 weeks of age; 160 ± 20 g) were purchased from the Egyptian Organization for Biological Products and Vaccines, Giza, Egypt. Rats were fed a regular standard diet and water ad libitum. Experiments were conducted in accordance with the ethical guidelines for investigations in laboratory animals and approved by the Ethical Committee of Faculty of Pharmacy, Mansoura University, Egypt (Code 2019-79).

### 2.6. GLE Preparation for Oral Administration to Rats (In Vivo Studies)

Dry GLE material was initially dissolved in minimal amount of absolute EtOH (<3% of the final volume), then diluted with distilled water to a final concentration of 25 g/100 mL.

### 2.7. Experiment Design

Rats were initially weighed and randomized into 5 groups (n = 6–8): CON, GLE, EtOH, GLE_250_/EtOH, and GLE_500_/EtOH. Rats in the CON and EtOH groups were administered the vehicle (distilled water containing < 3% absolute ethanol). Rats in GLE, GLE_250_/EtOH, and GLE_500_/EtOH were orally administered GLE at doses of 250 (GLE_250_/EtOH) or 500 mg/kg (GLE and GLE_500_/EtOH). Absolute EtOH was diluted to a 50% solution with distilled water and orally administered to rats in the EtOH, GLE_250_/EtOH, and GLE_500_/EtOH groups at a dose of 6 g/kg [11], 2 h after the extract. CON and GLE groups received distilled water instead. Experiment design is shown in Table 1. Previous studies showed hepatoprotective effects for different kinds of grape extracts at different dose levels (250–500 mg/kg) in different experiment models of liver injury [1,6,12]. The current study employed GLE at doses of 250 and 500 mg/kg, where both EtOH and GLE were administered for 12 d (which was shown by our preliminary experiment to induce a marked increase in all measured markers of liver injury (Supplementary Materials).

### 2.8. Collection of Serum, Plasma, and Tissue Homogenate

At the end of the experiments (day 12), rats were weighed, anesthetized, and blood samples were collected. Centrifugation (2000× *g*, 15 min, 4 °C) was then performed after the samples were allowed to clot for 45 min, and the retrieved serum was stored at −80 °C for further measurements. For plasma, blood was collected within tubes coated with heparin, and centrifugation (2000× *g*, 15 min, 4 °C) was immediately performed to separate the plasma. Livers were rapidly dissected, washed using ice-cold saline, and weighed. Liver coefficients were calculated according to the formula; $Liver\;coefficient\;\left(\%\right)=\frac{Liver\;weight}{Body\;weight}\times 100$ [13]. Left lobes were then fixed in neutral-buffered formalin for histopathological examination and immunohistochemical (IHC) analysis. Part of the right lobes were homogenized in ice-cold phosphate buffer (10% *w***/***v*, pH 7.4).

### 2.9. Assessment of Biochemical Markers of Liver Function

Bilirubin was measured spectrophotometrically in the plasma. Aspartate aminotransferase (AST), alanine aminotransferase (ALT), gamma-glutamyl transferase (GGT), alkaline phosphatase (ALK-p) and albumin were spectrophotometrically measured in serum using commercial kits (SPINREACT, Sant Esteve de Bas, Spain) in accordance to the given instructions.

### 2.10. Assessment of Total Nitrite/Nitrate (NOx) Products

NOx, an indicator of reactive nitrogen species (RNS), was measured according to a previously described method [14]. Briefly, 0.5 mL of liver homogenate was mixed with 0.25 mL of 0.3 N NaOH, and the reaction was incubated at room temperature for 5 min. This was followed by deproteinization using 0.25 mL of 5% (*w*/*v*) ZnSO4. The reaction mixture was centrifuged at 3000× *g* for 20 min at 4 °C. Then, 0.5 mL of the supernatant was mixed with 0.3 mL VCl3 (8 mg/mL) in 1M HCl and 0.3 mL Griess reagent composed of the following: 0.15 mL of 2% (*w*/*v*) sulphanilamide in 5% (*v*/*v*) HCl and 0.15 mL of 0.1% (*w*/*v*) N-(1- naphthyl)-ethylenediamine dihydrochloride in distilled water. The reaction mixture was incubated at 37 °C for 45 min, and the product was measured spectrophotometrically at 540 nm. NOx concentrations were computed from a calibration curve constructed using NaNO_3_ (0–100 nmol/mL).

### 2.11. Assessment of MDA and Superoxide Dismutase (SOD) Activity

Thiobarbituric acid reactive substance (TBARS) was measured as MDA, the end product of lipid peroxidation, according to the method previously described [15]. Briefly, 0.2 mL of liver homogenate was mixed with 0.2 mL of 8.1% sodium dodecyl sulphate, 1.5 mL of 20% acetic acid solution adjusted to pH 3.5 with NaOH and 1.5 mL of 0.8% aqueous solution of TBA. The reaction was incubated at 95 °C for 60 min followed by centrifugation at 2000× *g* for 10 min. Absorbance of the organic layer was measured at 532 nm.

SOD activity was assayed by the method previously described [16]. The reaction mixture was composed of 0.1 mL of liver homogenate with 1.5 mL of 20 mM Tris-HCl, 1 mM EDTA, pH 8.2 then 0.1 mL of 15 mM pyrogallol was added. The change of absorbance at 420 nm per minute was monitored over a three-minute period. Controls with no samples were run under the same conditions in order to compute the rate of inhibition. The enzyme activity was expressed as U/g where one unit represents the number of enzymes that suppresses pyrogallol autooxidation by 50%.

### 2.12. Assessment of Hepatic GSH Levels and Glutathione-Utilizing Enzyme Activities: Glutathione Reductase (GR) and Glutathione Peroxidase (GPx)

The levels of acid-soluble thiols, mainly reduced glutathione (GSH), were assayed as described previously [17]. We used 500 μL of 50% (*w*/*v*) TCA to precipitate the protein in liver homogenate. The supernatant was collected after centrifugation at 1000× *g* for 5 min and 0.1 mL of the supernatant was mixed with 1.7 mL phosphate buffer (0.1 M, pH 8) and 0.1 mL Ellman’s reagent. The reaction was kept at room temperature for 5 min. Yellow was formed and measured spectrophotometrically at 412 nm.

Activities of GR, the enzyme mainly responsible for glutathione reduction, and GPx, the enzyme responsible for reducing H_2_O_2_ into water with the consumption of GSH, were measured using kits (Biodiagnostics, Giza, Egypt).

### 2.13. Measurement of Proinflammatory Cytokines: Interleukin-6 (IL-6) and TNF-α Levels in Liver Homogenate

IL-6 and TNF-α levels were determined using ELISA kits from Thermo Scientific (Rockford, IL, USA) and AssayPro. (St. Charles, MO, USA), respectively, according to the manufacturer instructions.

### 2.14. Histopathological Examination of Liver Tissue

Paraffin-embedded liver tissue was used for sectioning (7 μm thick sections). Sections were then stained with hematoxylin and eosin (H&E) and examined using light microscope, and digital pictures were captured by a digital camera. Histopathological examination was performed by a single independent pathologist, blinded to the experiment design and treatment groups. Hepatic steatosis and inflammation were graded on a semiquantitative scale of 0–3, while ballooning degeneration was scored 0–2 as previously described by [18].

### 2.15. IHC Analysis

NF-κB p65, heat-shock protein-70 (Hsp-70), caspase-3, and survivin in liver-tissue sections were immunostained with the Avidin-Biotin Complex (ABC) method [19] using rat monoclonal antibodies (Thermo Fisher Scientific, CA, USA). The cross-sections were dewaxed, hydrated, and immersed in antigen retrieval (EDTA solution, pH 8). They were then treated with hydrogen peroxide 0.3% and blocked by goat serum (5%, 1 h), followed by incubation with primary antibody. The slides were then rinsed three times with phosphate-buffered saline, incubated with antirabbit IgG secondary antibodies (Envision system HRP, Dako) for 30 min at room temperature, and visualized with d-aminobenzidine commercial kits (liquid DAB+ substrate chromogen system; Dako).

### 2.16. Statistical Analysis

Values are represented as means ± SEM. All parameters were analyzed using one-way analysis of variance (ANOVA) followed by Tukey’s multiple-comparison test. The Kruskal–Wallis test, followed by Dunn’s multiple-comparison test, was used for histopathological scoring and immunohistochemical analysis. Values of *p* ≤ 0.05 were considered statistically significant. Statistical analysis was carried out using GraphPad Prism software (GraphPad Software Inc. V6.0c, San Diego, CA, USA).

## 3. Results

### 3.1. LC–MS Analysis

**Figure 1:**
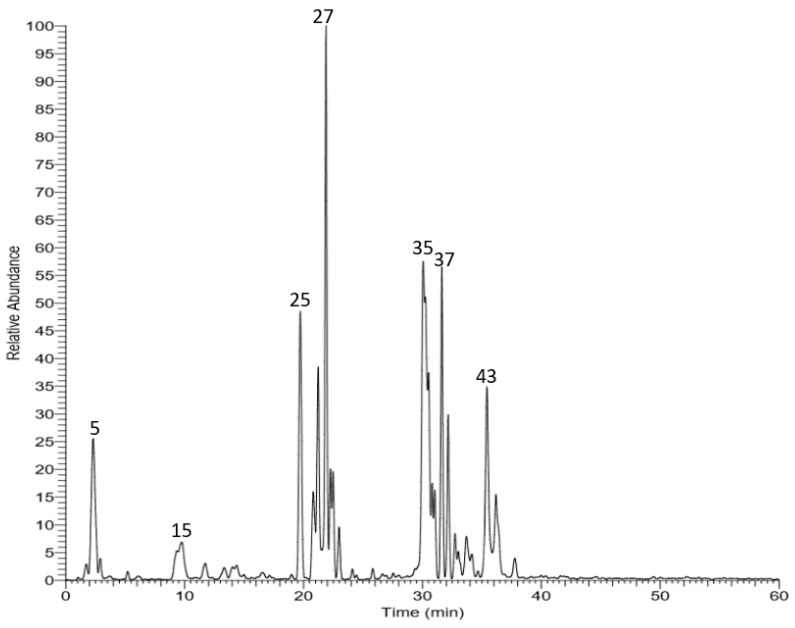
LC–MS profile of total methanol extract of grape leaves.

Different parts of *Vitis vinifera* have been extensively studied, especially the leaves, fruits, and seeds that provide one of the major sources of phenolic compounds [20]. However, only few reports exist on the leaves apart from detailed LC–MS analysis. In this work, HPLC–PDA–ESI–MS was carried out for the tentative identification of the phytoconstituents in the methanol extract of grape leaves. A total of forty-five secondary metabolites were tentatively identified. A phytochemical profile is shown in Figure 1. The detected and identified compounds are listed in Table 2 with the corresponding retention and MS/MS fragmentation data. Several anthocyanins, hydroxycinnamic acid derivatives, and flavonols have been reported from the leaves to characterize different commercial antioxidant dietary ingredients derived from the leaves and skins of grapes. In our study, the identified compounds belonged to different classes, e.g., anthocyanins, phenolic acids and derivatives, flavonoids, stilbenoids, and organic acids.

### 3.2. GLE Did Not Significantly Alter EtOH-Induced Body-Weight Increase

**Figure 2:**
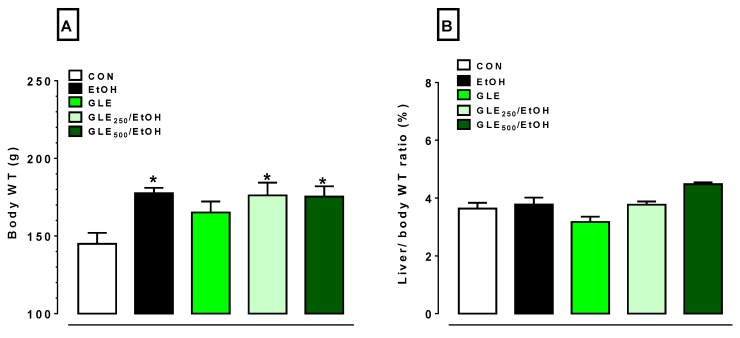
GLE effects on body weight and liver coefficient in EtOH-treated rats. Bar graphs show changes in (**A**) body weight and (**B**) liver coefficient. Liver coefficients were calculated according to formula of liver coefficient (%) = (liver weight)/(body weight) × 100. Data expressed as mean ± SEM (n = 5–8). * *p* < 0.05 compared to control using one-way analysis of variance (ANOVA) followed by Tukey’s multiple-comparison test. CON: control, EtOH: ethanol, GLE: grape leaf extract.

As shown in Figure 2, rats treated with EtOH showed a 1.4-fold increase in body weight compared to the control rats. Administration of GLE_250_ and GLE_500_ did not significantly affect EtOH-induced changes in body weight (Figure 2A). Moreover, the liver coefficient was not significantly altered in any group (Figure 2B).

### 3.3. GLE Markedly Attenuates EtOH-Induced Changes in Liver-Injury Markers

**Figure 3:**
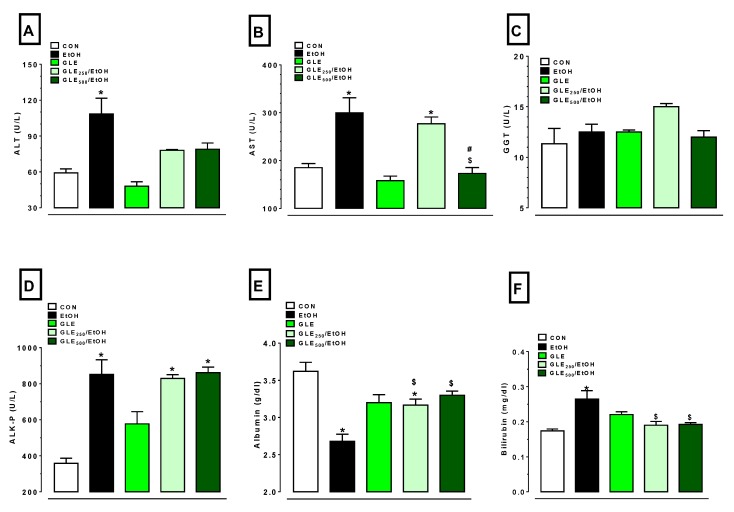
GLE effects on liver-injury markers in EtOH-treated rats. Bar graphs showing changes in (**A**) ALT, (**B**) AST, (**C**) serum GGT, (**D**) serum ALK-P, (**E**) serum albumin, and (**F**) plasma bilirubin. Data expressed as mean ± SEM (n = 4–6). ^*,$,#^ *p* ≤ 0.05 compared with CON, EtOH, GLE_250_/EtOH groups, respectively. Statistical analysis was performed using one-way ANOVA followed by Tukey’s multiple-comparison test. CON: control, EtOH: ethanol, GLE: grape-leaf extract, ALT: alanine aminotransferase; AST: aspartate aminotransferase; GGT: gamma-glutamyl transferase; ALK-p: alkaline phosphatase.

The increase in liver-injury markers is commonly seen in EtOH-induced liver dysfunction. In the experiment end (Day 12), EtOH caused significant increases in ALT (1.8-fold), AST (1.6-fold), ALK-p (2.4-fold), and bilirubin (1.5-fold), and a significant decrease in albumin (26% decrease) compared to the control. Administration of GLE_500_ caused 20% and 69.6% inhibition of EtOH-induced increases in AST and bilirubin levels, respectively (*p* ≤ 0.05). Administration of GLE_250_ caused inhibition of EtOH-induced increase by 64.9% in bilirubin levels (*p* ≤ 0.05), while AST levels were still significantly higher compared to both control and GLE_500_. Both GLE_250_ and GLE_500_ groups mediated a significant elevation of bilirubin (1.2-fold) compared to the EtOH group. ALT levels with GLE_250_ and GLE_500_ were not significantly different compared to thos of the control (or EtOH) groups. Changes in GGT were not significantly different between all groups (Figure 3A–F).

### 3.4. GLE Promotes Hepatic Antioxidant Defense in EtOH-Treated Rats

**Figure 4:**
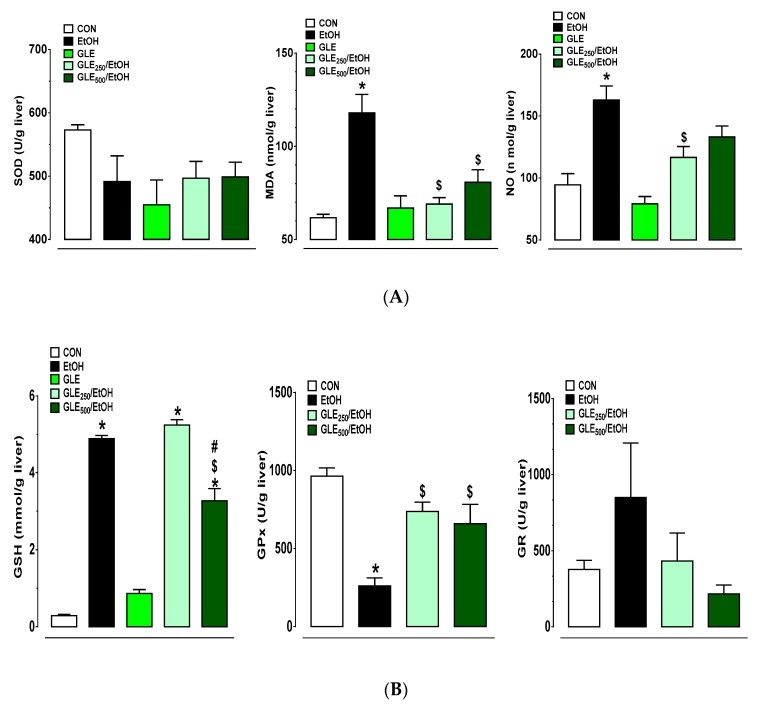
GLE effects on markers of oxidative stress in EtOH-treated rats. Bar graphs show changes in SOD (left panel), MDA (middle panel) and NO metabolites (right panel) (**A**) and GSH (left panel), GPx (middle panel) and GR (right panel) (**B**). Data expressed as mean ± SEM (n = 5–8), ^*,$,#^ *p* ≤ 0.05 compared with CON, EtOH, GLE_250_/EtOH group, respectively. Statistical analysis performed using one-way ANOVA followed by Tukey’s multiple-comparison test. CON: control, EtOH: ethanol, GLE: grape leaf extract, SOD: superoxide dismutase, MDA: malondialdehyde, NO: nitric oxide, GSH: reduced glutathione, GPx: glutathione peroxidase, and GR: glutathione reductase.

As shown in Figure 4A, EtOH administration caused 1.8- and 1.7-fold increases in TBARS (measured as MDA equivalents) and NO metabolites, respectively. GLE_250_ mediated 91% (*p* ≤ 0.05) and 67.7% (*p* ≤ 0.05) decreases in EtOH-induced increases in MDA and NO metabolites, respectively. GLE_500_ caused 69.3% (*p* ≤ 0.05) decrease in EtOH-induced increase in MDA. SOD changes were not statistically significant in any group. EtOH administration also resulted in a 73% decrease in Gpx activity with an 18.5-fold increase in GSH (*p* ≤ 0.05). A nonsignificant (1.2-fold) increase in GR activity was also noted with ethanol administration that could partly account for the recycling of glutathione into its reduced form (GSH), but the decrease in GPx activity may attenuate its radical scavenging function as previously observed [33]. GLE_250_ caused a 2.8-fold increase in GPx activity of EtOH groups, while GLE_500_ showed a 40.6% decrease in EtOH-induced increase in GSH and twofold increase in GPx activity compared to EtOH groups, respectively (*p* ≤ 0.05). All observed changes in GR activity were not statistically significant (Figure 4B).

### 3.5. GLE Decreases HSP-70 Expression in Liver of EtOH-Treated Rats

**Figure 5:**
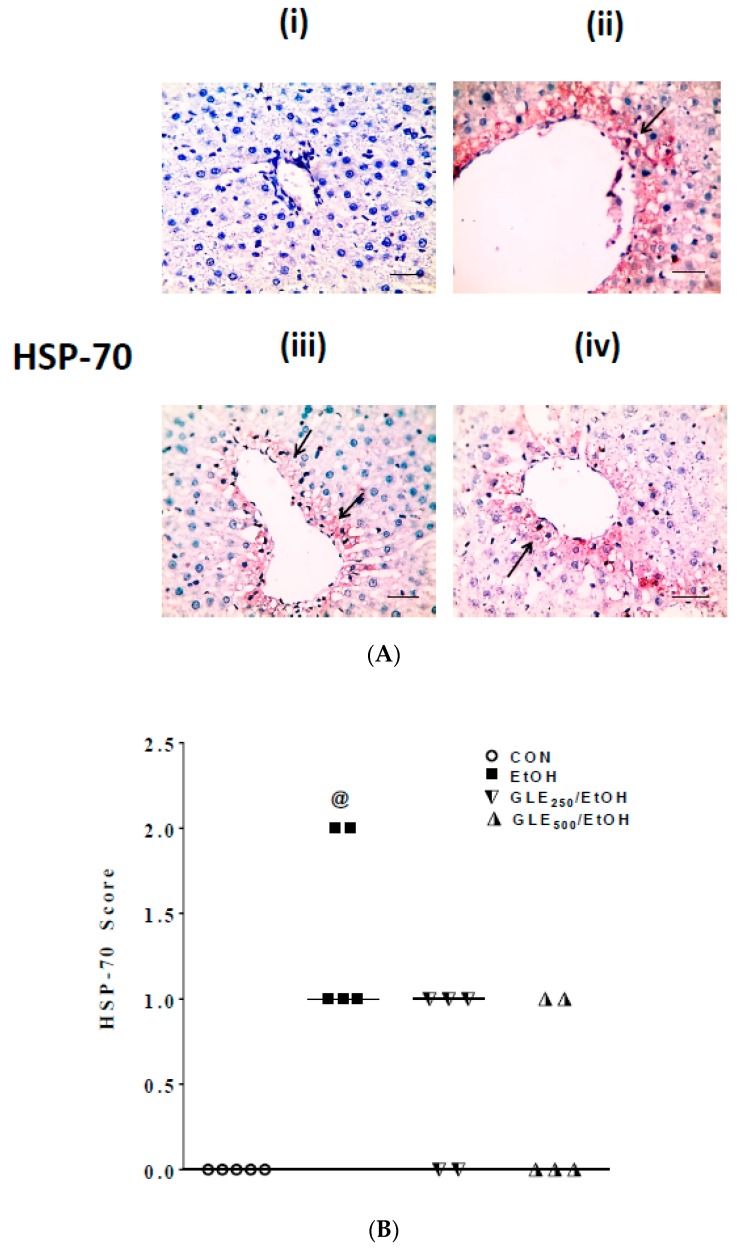
GLE effects on hepatic expression of HSP-70 in EtOH-treated rats. Representative photomicrographs of immunohistochemical detection of HSP-70 in livers from CON (i), EtOH (ii), GLE_250_/EtOH (iii) and GLE_500_/EtOH (iv) groups (**A**) and scatter-dot plot showing the score of HSP-70 (median is indicated by a line) in the same groups (**B**). ^@^ *p* ≤ 0.05 compared with CON, using Kruskal–Wallis followed by Dunn’s Multiple Comparison test (n = 5). CON: control, EtOH: ethanol, GLE: grape leaf extract, HSP-70: heat shock protein-70.

An increase in expression of HSP-70 is commonly seen in EtOH-induced injury [34]. As shown in Figure 5A,B, no immunoreactivity to HSP-70 was seen in control liver tissue (i, no brown color; score = 0), whereas moderate immunoreactivity to HSP-70 was noted in the EtOH group in centrilobular areas (ii, arrows indicate areas of positive expression of HSP-70, score = 1–2). Weak positive staining for HSP-70 was found in the centrilobular areas in GLE_250_ (iii) and GLE_500_ (iv) livers (score = 0–1). Images were taken with 400 magnification and 50 µm scale bar.

### 3.6. GLE Enhances Anti-Inflammatory Activity in Liver of EtOH-Treated Rats

**Figure 6:**
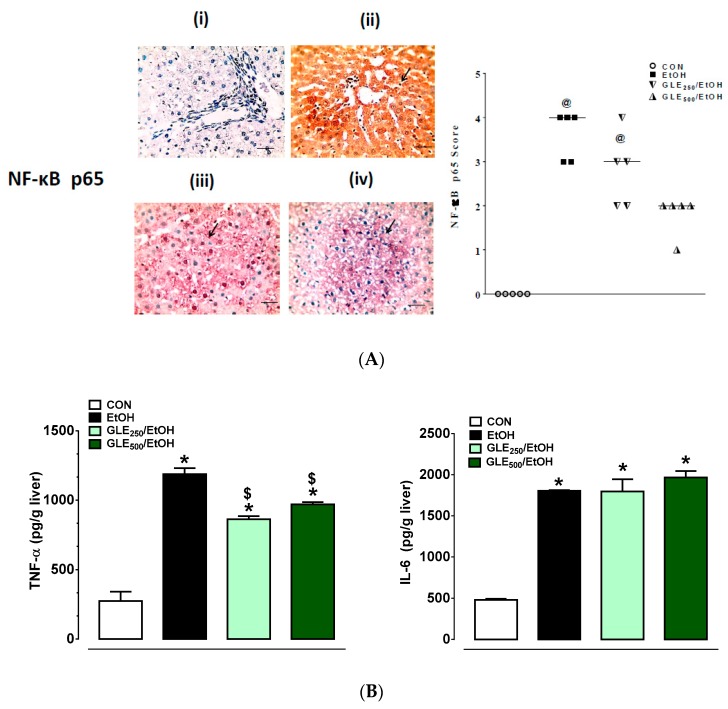
GLE effects on hepatic expression of NF-κB p65 subunit and proinflammatory cytokines in EtOH-treated rats. Representative photomicrographs of immunohistochemical detection of NF-κB p65 in livers from CON (i), EtOH (ii), GLE_250_/EtOH (iii), and GLE_500_/EtOH (iv) groups (left panel, **A**), scatter-dot plot showing the score of NF-κB p65 (median is indicated by a line) in the same groups (right panel, **A**), bar graphs showing changes in TNF-α (left panel, **B**) and IL-6 (right panel, **B**). Data expressed as mean ± SEM in bar graphs (n = 5). ^@^ *p* ≤ 0.05 compared with CON group (right panel, A), and ^*,$,#^ *p* ≤ 0.05 compared with CON, EtOH, GLE_250_/EtOH group, respectively (**B**). Statistical analysis was performed using Kruskal–Wallis followed by Dunn’s multiple-comparison test (right panel, A) or one-way ANOVA, followed by Tukey’s multiple-comparison test (**B**). CON: control, EtOH: ethanol, GLE: grape-leaf extract, NF-κB: nuclear factor NF-κB, TNF-α: tumor necrosis factor-α, IL-6: interleukin 6.

Previous studies showed that inflammatory markers, especially NF-κB p65, TNF-α, and Il-6, play a role in EtOH-induced inflammation [34]. As shown in Figure 6A, no immunoreactivity to NF-κB p65 was seen in the control liver tissue (i, score = 0); while strong positive staining to NF-κB p65 was seen in livers from the EtOH group, particularly in centrilobular and periportal areas (ii, score = 3–4). The intensity of staining slightly decreased in GLE_250_/EtOH group (iii, score = 2–3), with a greater extent of decreased immunoreactivity seen in GLE_500_/EtOH group (iv, score = 1–2). Images were taken with 400 magnification and 50 µm scale bar. TNF-α (Figure 6B, left panel) and Il-6 (Figure 6B, right panel) were increased by 4.3- and 3.7-fold, respectively, in EtOH compared to control group (*p* ≤ 0.05). GLE_250_ and GLE_500_ caused 35.7% and 23.9% decreases in EtOH-induced increase in TNF-α, respectively (*p* ≤ 0.05). Changes observed in Il-6 by GLE were not statistically significant.

### 3.7. GLE Enhances Antiapoptotic Activity in Liver of EtOH-Treated Rats

**Figure 7:**
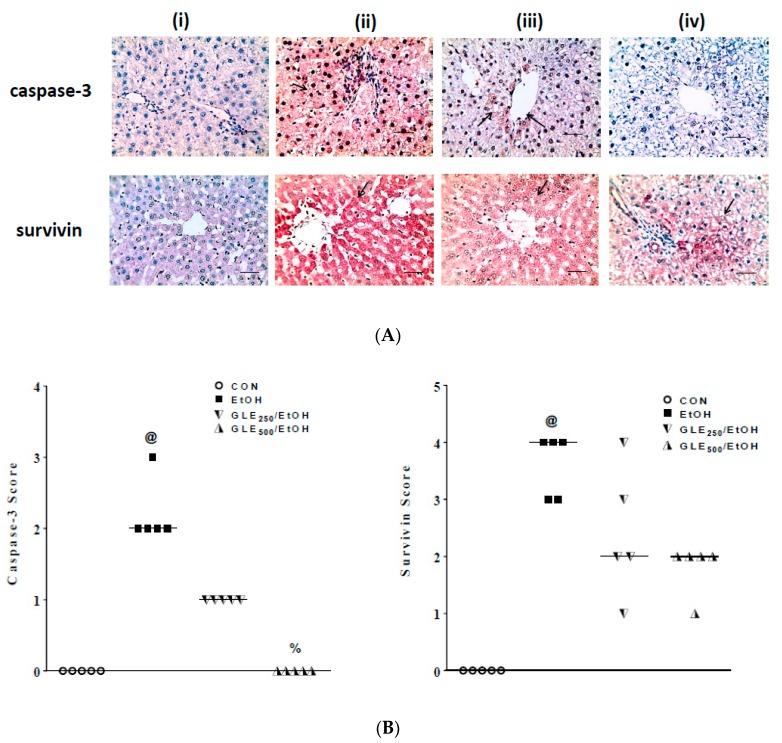
Effects of GLE on hepatic expression of caspase-3 and survivin in EtOH-treated rats. Representative photomicrographs of immunohistochemical detection of caspase-3 and survivin in livers from CON (i), EtOH (ii), GLE_250_/EtOH (iii) and GLE_500_/EtOH (iv) groups (**A**). Scatter-dot plot showing the score of caspase-3 (left panel) and survivin (right panel) in the same groups (**B**). Median is indicated by a line (**B**). ^@,%^ *p* ≤ 0.05 compared with CON and EtOH groups, respectively. Statistical analysis was performed using Kruskal–Wallis followed by Dunn’s multiple-comparison test (n = 5). CON: control, EtOH: ethanol, GLE: grape leaf extract.

An increase in apoptotic markers is commonly seen in EtOH-induced liver injury [35]. As shown in Figure 7A (upper panel) and B (left panel), no immunoreactivity to caspase-3 was seen in the control liver tissue (i, score = 0), while strong positive staining to caspase-3 was noted in the centrilobular and periportal areas (ii, score = 2–3) in EtOH group. Weak positive staining in centrilobular areas was observed in GLE_250_/EtOH group (iii, score = 1), while no immunoreactivity to caspase-3 was observed in GLE_500_/EtOH group (iv, score = 0).

As shown in Figure 7A (lower panel) and Figure 7B (right panel), no immunoreactivity to survivin was seen in the control liver tissue (i, score = 0), while strong positive staining to survivin particularly in centrilobular areas (ii, score = 3–4) was seen in EtOH group. The intensity of staining slightly decreased in GLE_250_/EtOH group (iii, score = 1–4), and strongly decreased in GLE_500_/EtOH group (iv, score = 1–2). Images were taken with 400 magnification and 50 µm scale bar.

### 3.8. GLE Diminished EtOH-Induced StructuralLiver Alteration

**Figure 8:**
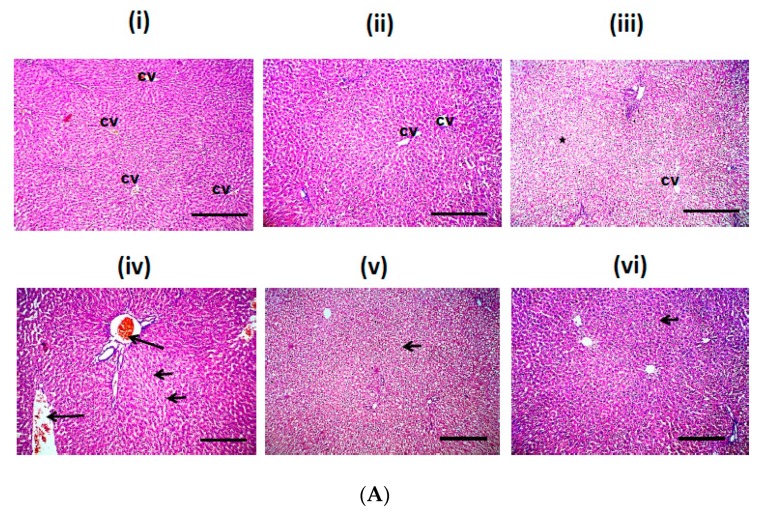

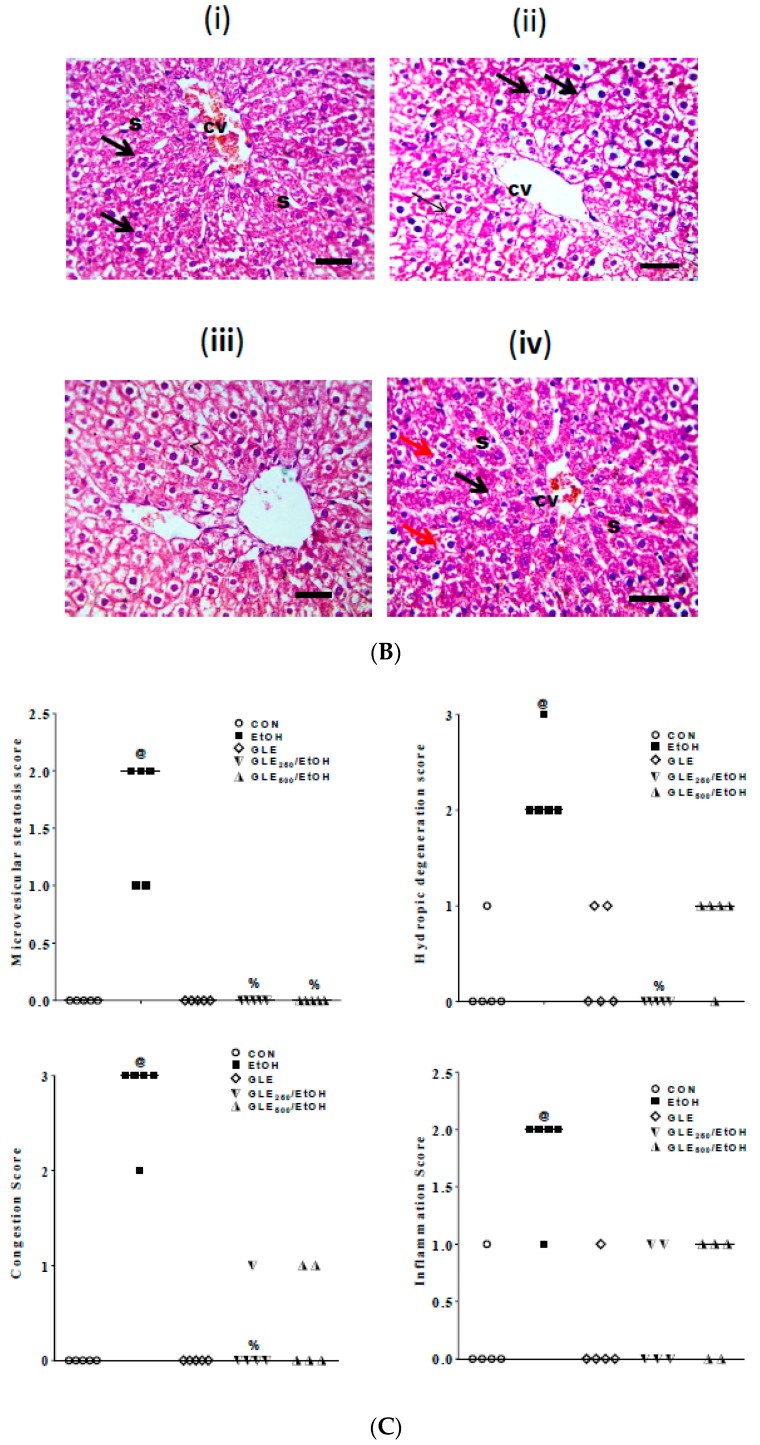
Effects of GLE on histopathological changes in liver sections in EtOH-treated rats. (**A**) Representative photomicrographs of liver sections stained with H&E from CON (i), EtOH (ii–iv), GLE_250_/EtOH (v) and GLE_500_/EtOH (vi) groups; (**B**) from CON (i), EtOH (ii), GLE_250_/EtOH (iii) and GLE_500_/EtOH (iv) groups; (**C**) Scatter-dot plot showing microvesicular steatosis, hydropic degeneration, congestion and inflammation Score. The data shown are the median (n = 5), ^@,%^ *p* ≤ 0.05 compared with Control, EtOH, group, respectively. Statistical analysis was performed using Kruskal–Wallis followed by Dunn’s Multiple Comparison test.

EtOH exposure commonly results in liver damage, marked mixed micro- and macrovesicular steatosis, ballooning degeneration in the hepatocyte, and infiltration of mononuclear inflammatory cells in the livers [36]. As shown in Figure 8A, microscopic pictures of liver sections from control group (i) and GLE (ii) groups show radially arranged hepatic cords around the central vein (cv) and separated by adjacent blood sinusoids. Microscopic pictures of liver from EtOH group show partial loss of the normal cord like arrangement of the hepatic cords (asterisk, iii), markedly congested blood vessels (long arrows, iv), microvesicular steatosis in hepatocytes (short arrows, iv), with dilated congested intervening blood sinusoids and shrunken hepatic cords (iv). Livers from GLE_500_/EtOH show only diffuse hydropic degeneration of hepatocytes (short arrows) (v), while livers from GLE_250_/EtOH show mild hydropic degeneration of hepatocytes (short arrows, vi). Images were taken with magnification 100 and scale bar 100 µm.

As shown in Figure 8B, microscopic pictures of livers from the EtOH group showed congested central veins, swelling of hepatocytes due to hydropic degeneration (arrows), and narrowed blood sinusoids (i), or severe diffuse ballooning degeneration in hepatocytes with apoptotic nuclei (arrows) and obliterated blood sinusoids in some other sections (ii). Livers from GLE_500_/EtOH show diffuse severe hydropic degeneration of hepatocytes (short arrows, iii). Livers from GLE_250_/EtOH show mild hydropic degeneration of hepatocytes (short arrows), mild ballooning degeneration on the periphery of the hepatic lobules (red arrows) with narrowed blood sinusoids (iv). Images were taken with 400 magnification and 50 µm scale bar. Figure 8C shows that scores of microvesicular steatosis, hydropic degeneration, congestion, and inflammation in EtOH groups were significantly higher than those in the control group. Treatment with GLE at 250–500 mg/kg/d reduced the scores of these parameters.

## 4. Discussion

The current study tested two different doses of GLE in a model of EtOH-induced liver damage to investigate the mechanisms associated with the hepatoprotective effects of GLE. GLE attenuated liver injury (↓ AST, ↓ plasma bilirubin and ↑ serum albumin) induced by oral administration of high doses of EtOH. This improvement in markers of liver damage was associated with marked hepatic antioxidant effects (↓ lipid peroxidation, normalized GSH level and Gpx activity and ↓ RNS metabolites (NO_2_/NO_3_)). Changes in hepatic expression of HSP-70 that accompanied EtOH-induced oxidative stress were accordingly attenuated by GLE. Moreover, GLE suppressed EtOH-induced expression of NF-κB p65 subunit and proinflammatory cytokine TNF-α. Hepatic apoptosis, along with antiapoptotic markers, were stimulated by EtOH intake and suppressed by GLE intake (↓ caspase-3 and survivin). Finally, EtOH-induced histopathological changes in liver sections were markedly normalized by GLE (↓ inflammation, ↓ microvesicular steatosis, ↓ hydropic degeneration and ↓ congestion scores). Our results suggested that GLE interferes with NF-κB signaling and induces antioxidant effects, which both play a role in attenuating apoptosis and associated liver injury in a model of EtOH-induced liver damage in rats.

In the current study, oxidative stress was developed in EtOH-treated rats (↑ lipid peroxidation and RNS metabolite accumulation). Those results support previous well-accepted EtOH metabolism-induced oxidative stress and increased ROS that are known to play an important role in EtOH-induced hepatotoxicity [2,37]. Additionally, despite the observed increase in GSH in EtOH-treated rats in the current study, which could be accounted for by the increase in GR activity and increased recycling of glutathione into its reduced form, but the decrease in GPx activity would eventually attenuate its radical scavenging function, as previously observed [33]. GLE could normalize all previously mentioned oxidative stress markers, and normalize HSP-70, which has been closely linked to hepatic oxidative stress [34], further supporting its antioxidant effects in this model and in accordance to what was shown by others in a model of chronic EtOH administration in rats [11]. SOD activity was not changed by EtOH administration in the current study, which was in accordance with other results [38,39], which could be due to a defense mechanism initiated by the liver. The antioxidant effects observed for GLE (current study) could be partly attributed to the activation of nuclear factor erythroid 2 related factor 2 (Nrf2), a master transcription factor for antioxidant response, reported to be mediated by the secondary metabolites (e.g., apigenin derivative [5,40], epicatechin [41], quercetin derivatives [3,42], caffeic acid [43,44], rosmarinic acid derivatives [45,46], and isorhamnetin derivatives [4,47]) present in the extract. Future studies are needed to clearly address the effect of GLE on Nrf2 activation.

Under conditions of EtOH-induced hepatotoxicity, the interplay between EtOH metabolism-induced oxidative stress and EtOH-activated inflammatory responses is inevitable to hepatic injury and ALD development. In particular, oxidative stress and ROS generated with EtOH intake increase accumulation of lipids in hepatocytes, and sensitize the liver to both gut-derived endotoxin/lipopolysaccharide (LPS) and to subsequent insults by cytokines, as reviewed in [48]. Subsequently, alcohol directly or through LPS-activated toll-like receptors induces a cascade of events that end up with NF-κB activation and the production of proinflammatory cytokines, e.g., TNF-α and IL-6. NF-κB-induced TNF-α production is also implicated in fatty-liver development by EtOH [49]. Surprisingly, antioxidant treatments as single therapy did not meet the expectations in attenuating the different stages of EtOH-induced liver disease [50,51], further highlighting the importance of investigating the effects of any tested agent on EtOH-induced inflammation in the hope of finding more successful agents that could alleviate alcoholic liver disease in patients. Interestingly, GLE in the current study was found to exert both anti-inflammatory (↓ NF-κB/TNF-α/IL-6) and antioxidant effects.

Another major component that associates all forms of liver injury is apoptosis, which could be triggered by both NF-κB/inflammatory cytokines (e.g., TNF-α) and by excessive intracellular ROS, leading to activation of caspases (e.g., caspase 3) and subsequent cell death. Interestingly, apoptosis causative factors are able not only to induce apoptosis, but also to simultaneously stimulate survival signals (e.g., survivin) against cell death [52,53], with an end result (cell death or survival) depending on the severity of the initial insult [54]. The EtOH (current study) caused an increased expression of both apoptotic (caspase-3) and antiapoptotic (survivin) markers, and those changes were alleviated by GLE, which correlated well with its hepatoprotective effects demonstrated in the current study. Importantly, grape-seed extract was previously found to exert neuro-, cardio-, and renoprotective effects via apoptosis suppression [55,56], with no previous reports on the antiapoptotic effects of GLE in the liver.

In the current study, the identified compounds in GLE belong to different classes of natural products, e.g., anthocyanins, phenolic acids and derivatives, flavonoids, stilbenoids, and organic acids. Flavonoids were previously reported to exert a protective effect against alcohol-induced liver damage [57,58,59]. Anthocyanins, as a subclass of flavonoids, were previously found to play a role in the prevention of oxidative damage in living systems. The potential protective effect of GLE is probably mediated by the presence of such phenolic constituents which can interact with most proteins by forming hydrogen and ionic bonds [60], thus resulting in such biological activities. Future cell-based studies could further confirm changes observed in the current study.

## 5. Conclusions

The current study demonstrated the hepatoprotective effects of GLE against alcohol intoxication. In addition to the antioxidant effect, hepatoprotection was associated with the suppression of NF-κB expression and signaling to inflammatory cytokines, both of which ended up with attenuated apoptosis in liver tissue and improved markers of liver injury along with marked improvement in histological liver derangements. The activity was mediated by different constituents identified in the extract, especially polyphenols that can interact with most proteins by forming hydrogen and ionic bonds, thus resulting in such biological activities.

## Figures and Tables

**Table 1:** Experiment design to determine effect of grape-leaf extract (GLE) on ethanol (EtOH)-induced liver injury.

	CON	GLE	EtOH	GLE_250_/EtOH	GLE_500_/EtOH
Ethanol (6g/kg, orally)			√	√	√
Distilled water (vehicle for ethanol)	√	√			
GLE (250 mg/kg, orally)				√	
GLE (500 mg/kg, orally)		√			√
Distilled water containing < 3% absolute ethanol (vehicle for GLE)	√		√		

**Table 2:** Chemical composition of methanol extract of grapes leaves.

No.	*t_R_* (min)	[M-H]^-^	MS/MS Fragments	Tentatively Identified Compound	Ref.
1	1.03	149	59, 87, 102	Tartaric acid	[21]
2	1.70	191	111, 173	Citric acid	[21]
3	1.79	133	115	Malic acid	[22]
4	1.88	311	149, 179	Caftaric acid	
5	2.69	295	149, 163	Coutaric acid	
6	2.92	325	193	Fertaric acid	
7	3.28	299	137	*p*-hydroxybenzoic acid-*O*-hexoside	
8	3.84	331	125, 169	Monogalloyl glucose	[23]
9	4.94	315	153	Protocatechuic acid-*O*-hexoside	
10	5.52	341	135, 179	Caffeic acid-*O*-hexoside	[24]
11	5.72	164	147	Phenylalanine	[25]
12	5.82	315	153	Protocatechuic acid-*O*-hexoside	
13	6.18	509	347	Unknown	
14	7.39	503	179, 297, 341	Caffeic acid-*O*-dihexoside	[26]
15	9.74	341	135, 161, 179	Caffeic acid-*O*-hexoside	[24]
16	11.29	577	289, 407, 451	B-type procyanidin dimer	[23]
17	13.68	865	289, 407, 577	B-type procyanidin trimer	
18	13.90	639	301, 463, 477	Quercetin *O*- glucosyl glucuronide	
19	14.17	289	179, 205, 245	Catechin	[27]
20	14.42	865	287, 407, 425, 577	B-type procyanidin trimer	
21	15.23	289	179, 205, 245	Epicatechin	[27]
22	15.65	625	301, 463	Quercetin-*O*-hexoside-*O*-hexoside	[24]
23	17.19	625	463	Quercetin-*O*-hexoside-*O*-hexoside	[24]
24	17.57	521	359	Rosmarinic acid-*O*-hexoside	[28]
25	19.45	521	359	Rosmarinic acid-*O*-hexoside	[28]
26	20.59	477	151, 179, 301	Quercetin glucuronide	[29]
27	21.83	477	151, 179, 301	Quercetin glucuronide	[29]
28	22.94	477	179, 301, 315	Isorhamnetin-*O*-hexoside	[30]
29	23.48	625	463	Quercetin-*O*-hexoside-*O*-hexoside	[24]
30	25.85	477	301, 315	Isorhamnetin-*O*-hexoside	[30]
31	26.10	479	179, 316, 317	Myricetin-*O*-hexoside	[30]
32	26.63	477	301, 315	Isorhamnetin-*O*-hexoside	[30]
33	27.49	595	179, 271, 301	Quercetin-*O*-pentoside-*O*-hexoside	[27]
34	27.54	505	300, 301, 463	Quercetin-*O*-(acetyl)hexoside	
35	30.88	609	179, 271, 301, 463	Quercetin-*O*-rhamnoside-*O*-hexoside	[31]
36	31.48	463	179, 255, 271, 301	Quercetin-*O*-hexoside	[31]
37	32.67	463	179, 255, 271, 301	Quercetin-*O*-hexoside	[31]
38	32.79	463	179, 271, 301	Quercetin-*O*-hexoside	[31]
39	33.05	433	151, 179, 301	Quercetin-*O*-pentoside	[31]
40	33.63	447	284, 285, 327	Kaempferol-*O*-hexoside	[32]
41	35.17	623	300, 315	Isorhamnetin-*O*-coumaroyl-*O*-hexoside	
42	35.27	447	284, 285	Kaempferol-*O*-hexoside	
43	35.49	389	227	Resveratrol hexoside	
44	36.53	433	301	Quercetin-*O*-pentoside	
45	37.37	447	284, 300, 301	Quercetin-*O*-rhamnoside	

## Supplementary Materials

The following are available online at https://www.mdpi.com/2218-273X/10/4/558/s1, Figure S1: Effects of EtOH on markers of liver injury at different time points.

## Abbreviations

HPLC–PDA–ESI-MS	high-performance/pressure liquid chromatography–photodiode-array–electrospray ionization–mass spectrometry
ALK-p	alkaline phosphatase
ALT	alanine aminotransferase
AST	aspartate aminotransferase
CON	control
EtOH	ethanol
GGT	gamma-glutamyl transferase
GLE	grape leaf extract
GPx	glutathione peroxidase
GR	glutathione reductase
GSH	reduced glutathione
MDA	malondialdehyde
NF-κB	nuclear factor-κB
NO	nitric oxide
RNS	reactive nitrogen species
SOD	superoxide dismutase
TNF-α	tumor necrosis factor-α
