# Supplementary material for: Grape-Leaf Extract Attenuates Alcohol-Induced Liver Injury via Interference with NF-κB Signaling Pathway
Source: Biomolecules. 2020 Apr 6;10(4):558. doi: 10.3390/biom10040558 (PMC7225955; doi:10.3390/biom10040558)
Supplement: Supplementary file 1 [file biomolecules-10-00558-s001.pdf]

Supplementary data

Methods:

Experimental design

Five male Sprague Dawley rats were orally administered with ethanol (EtOH, 50%, 6 g/kg/day) for 12 days. Rats were anesthetized using thiopental (40 mg/kg, IP) and blood samples were collected from retro-orbital plexuses initially and every 4 days (i.e.; at 0, 4, 8, and 12 days). Centrifugation (2000 g, 15 min, 4 ^{0}C) was then performed after the samples were allowed to clot for 45 min, and the retrieved serum was stored at -80 ^{0}C for further measurements.

Assessment of biochemical markers of liver function in serum

Serum glutamic oxaloacetic transaminase (sGOT), serum glutamic pyruvic transaminase (sGPT), gamma-glutamyl transferase (GGT), alkaline phosphatase (ALK-p) and albumin were measured spectrophotometrically in serum using commercial kits (SPINREACT, Sant Esteve de Bas, Spain) in accordance with the provided instructions.

Results

EtOH-induced changes in liver markers.

As shown in Fig 1, EtOH caused a significant increase in sGPT starting at day 4 (compared with day 0) of EtOH intake. Moreover, EtOH caused significant increases in sGOT, ALK-P and GGT starting at days 8 (sGOT and ALK-P) or 12 (GGT) of intake. Albumin was significantly decreased only at day 12 of EtOH intake.

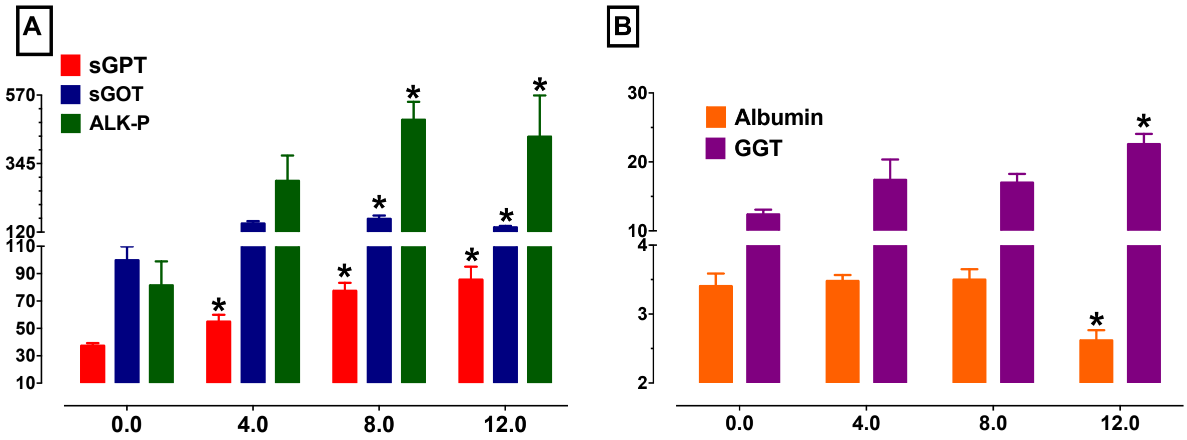

Figure 1: Effects of EtOH on markers of liver injury at different time points. The data shown are the means ± SEM (n=5). $^{*}p \leq 0.05$ compared with day 0. Statistical analysis was performed using one-way analysis of variance test (ANOVA) followed by Tukey's multiple comparison test for each marker separately. sGPT: Serum glutamic pyruvic transaminase; sGOT: Serum glutamic oxaloacetic transaminase; ALK-P: Alkaline phosphatase; GGT: Gamma-glutamyl transferas.
